# Validation of the diagnostic efficacy of O-RADS in adnexal masses

**DOI:** 10.1038/s41598-023-42836-1

**Published:** 2023-09-21

**Authors:** Na Su, Ya Yang, Zhenzhen Liu, Luying Gao, Qing Dai, Jianchu Li, Hongyan Wang, Yuxin Jiang

**Affiliations:** Department of Ultrasound, Peking Union Medical College Hospital, Chinese Academy of Medical Sciences and Peking Union Medical College, No.1 Shuai Fu Yuan, Dong Cheng District, Beijing, 100730 China

**Keywords:** Gynaecological cancer, Ovarian cancer, Cancer

## Abstract

The aim of this study was to validate the performance of the Ovarian-Adnexal Reporting and Data Systems (O-RADS) series models proposed by the American College of Radiology (ACR) in the preoperative diagnosis of adnexal masses (AMs). Two experienced sonologists examined 218 patients with AMs and gave the assessment results after the examination. Pathological findings were used as a reference standard. Of the 218 lesions, 166 were benign and 52 were malignant. Based on the receiver operating characteristic (ROC) curve, we defined a malignant lesion as O-RADS > 3 (i.e., lesions in O-RADS categories 4 and 5 were malignant). The area under the curve (AUC) of O-RADS (v2022) was 0.970 (95% CI 0.938–0.988), which wasn’t statistically significantly different from the O-RADS (v1) combined Simple Rules Risk (SRR) assessment model with the largest AUC of 0.976 (95% CI 0.946–0.992) (*p* = 0.1534), but was significantly higher than the O-RADS (v1) (AUC = 0.959, *p* = 0.0133) and subjective assessment (AUC = 0.918, *p* = 0.0255). The O-RADS series models have good diagnostic performance for AMs. Where, O-RADS (v2022) has higher accuracy and specificity than O-RADS (v1). The accuracy and specificity of O-RADS (v1), however, can be further improved when combined with SRR assessment.

## Introduction

Most ovarian cancers are in advanced stages when detected, and the prognosis for ovarian cancers detected in advanced stages is relatively poor compared to earlier stages^1,2^. Given the benignity and malignancy and stage of adnexal masses (AMs), the treatment options available to clinicians are quite different^3^. Therefore, timely and accurate preoperative assessment of the benignity and malignancy of AMs is extremely important and necessary.

Transvaginal ultrasound (US) is the preferred method of examination for AMs, but its role in scanning for AMs is not maximized due to the lack of a standardized examination method and standardized report descriptions^3,4^. It is now generally accepted that the subjective assessment by an experienced sonographer is more accurate in assessing the benignity or malignancy of AMs based on relevant clinical information^5,6^. However, this type of sonographer is not universally available in clinical practice. In order to improve the accuracy of ultrasonography and to achieve mutual recognition of reports, the International Ovarian Tumor Analysis (IOTA) group and the American College of Radiology (ACR) have successively proposed a series of diagnostic models for AMs^7–9^.

The IOTA Simple Rules (SRs) are a simple and clinically useful US rule to differentiate between benign and malignant AMs, which includes five benign and five malignant features and classifies lesions into benign, indeterminate and malignant categories accordingly^9–11^. To better predict the benignity and malignancy of the various types of AMs in the SRs and to further address the problem of grouping indeterminate categories, the IOTA working group developed the Simple Rules Risk (SRR) assessment model in 2016^12^. The model shares the same assessment conditions as the SRs and when the presence or absence of the 10 benign and malignant features from the SRs are entered into the website (available at SSRISK-model (kuleuven.be)), the system automatically generates a risk ratio for the lesion.

Based on the Ovarian-Adnexal Reporting and Data Systems (O-RADS) US Lexicon published in 2018, the ACR formally published the O-RADS version 1 (v1) US risk stratification and management system^7^. And so far O-RADS US version 2022 (v2022) has been released to rectify some of the deficiencies. This system classifies AMs into six categories from 0 to 5, covering all types of lesions from normal to highly malignant risk categories. In addition, the guideline also sets out management strategies for each risk category to standardize the clinical management of patients with different lesion categories.

From the introduction of O-RADS to its widespread use in the clinic, a large number of studies are still needed to validate its diagnostic efficacy. This study aims to validate the diagnostic efficacy of the O-RADS series models by comparing them with the subjective assessment of experienced sonologists. We also combined the O-RADS (v1) with the SRR assessment model in the course of the study, intending to improve the diagnostic specificity of the O-RADS (v1).

## Materials and methods

### Patients

**Figure 1:**
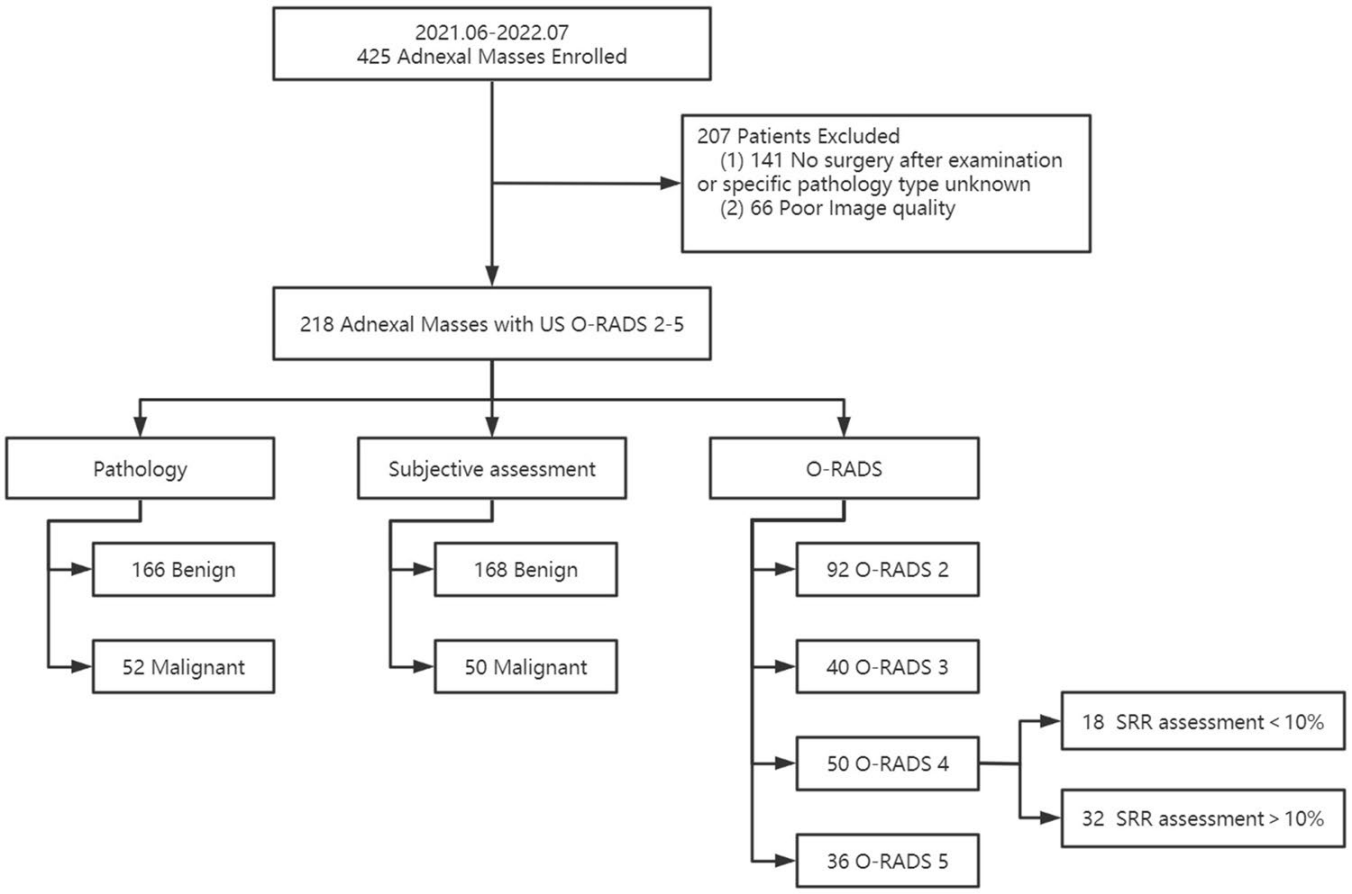
Flow chart for study population selection.

This prospective study was approved by the ethics committee of our hospital (Peking Union Medical College Hospital). All experiments were performed in accordance with relevant guidelines and regulations and all patients undergoing the examination have signed informed consent.We conducted a prospective study of 425 patients with AMs seen at Peking Union Medical College Hospital between June 2021 and July 2022. The inclusion criteria for this study were patients who presented with AMs (detected on imaging or clinical palpation). If patients had multiple lesions at the same time, we selected only one of the most suspicious lesions. The exclusion criteria for this study were as follows: (1) no surgical treatment after US or no specific type of pathology was provided; (2) failed image quality audit. We ultimately included 218 lesions from 218 patients. The flow chart of the study is shown in Fig. 1.

### Ultrasound examinations

US examinations were performed using Nuewa R9 equipment (Myriad Medical, Shenzhen, China). All ultrasound examinations were performed by two of the authors (N.S., Y.Y.), both of whom are sonologists in the Gynecology Specialty Group. Both sonologists received training on the O-RADS lexicon and O-RADS guidelines prior to the start of the study.

Transvaginal US was the preferred modality, but we used transabdominal US if the patient was unable to undergo transvaginal US or if the lesion was too large. Of the 218 patients, two patients underwent transabdominal US due to non-sexuality, 58 patients underwent transabdominal combined with transvaginal US due to the large size of the lesion (transabdominal US was used to measure the overall extent of the lesion and transvaginal US was used to observe the details of the lesion and blood flow), and the remaining patients underwent transvaginal US only. During the examination, both sonologists were required to capture images of the lesion: (1) greyscale images of the largest transverse and longitudinal planes of the lesion with and without measurement markers; (2) Color Doppler images of the most abundant blood flow; and (3) images of suspicious signs of the lesion, such as the presence of papillae and ascites. All images were reviewed by a sonologist with more than 15 years of experience in gynecology at our hospital. Images ultimately included in the study must meet the following criteria: (1) The images had to be clear; (2) The images were acquired according to the above requirements.

Once the examination was completed, the two sonologists were required to together give a subjective assessment of the lesion based on their experience and collaborated to give the lesion an O-RADS (v1) category based on the O-RADS lexicon and guidelines. The above assessments were given based on the characteristics of the lesions included in the studies. Due to the large malignancy span (10–50%) of O-RADS category 4, similar to the inclusive category in the IOTA SRs^10,11^, we further categorized lesions classified as O-RADS (v1) category 4 using the SRR assessment model, and in this study, a malignancy rate of 10% was used as the cut-off value for SSR to distinguish benign and malignant lesions^3,12^. For O-RADS category 4 lesions, the corresponding SRR assessment malignancy rate was further calculated and those lesions with less than 10% malignancy were downgraded to category 3. In addition, these 2 sonologists retrospectively analysed the images and gave a revised O-RADS classification based on the terminology described during the examination and the O-RADS US (v2022). If there was a disagreement between the two sonologists during the assessment, the senior sonologist responsible for the review decided the final outcome.

### Gold standard

The pathological diagnosis of surgically excised tissue was used as the gold standard during the study, and the tumors were classified according to the World Health Organization's International Classification of Ovarian Neoplasms^13^. As borderline tumors require surgical intervention as much as malignant tumors, they were classified as malignant in the course of this study^10^.

### Data analysis

SPSS 25.0 (IBM Corporation, Armonk, NY) and MedCalc 20.022 (MedCalc Software, Ostend, Belgium) software were used for statistical analysis during the study. Continuous variables were expressed as mean ± standard deviation, and categorical variables were expressed as frequencies. Comparisons of continuous variables were assessed using unpaired t-tests, and comparisons of categorical variables were made using chi-square tests and Fisher's exact tests. The receiver operating characteristic (ROC) curves were applied to calculate and compare the area under the curve (AUC) and to determine the best cut-off value. *p* < 0.05 was considered to be statistically significant.

## Results

### Clinical and image characteristics of the lesion

We studied 218 lesions in 218 patients with 166 benign and 52 malignant lesions with clinical and image characteristics as shown in Table 1. The mean age of the 218 patients was 44.78 ± 13.72 years (range 16–80 years). Among them, patients with benign lesions were younger than those with malignant lesions (*p* < 0.001). And, the maximum diameter of benign lesions was significantly smaller than that of malignant lesions (*p* < 0.001). There were also statistically significant differences in the O-RADS category and blood flow scores between benign and malignant lesions (all *P* < 0.001). The pathological types of lesions were shown in Table 2.

**Table 1:** Clinical and image characteristics of the 218 lesions.

			B (%)	M (%)	Total	P
Age			41.92 ± 12.64	52.13 ± 13.68		< 0.001
Maximum diameter of lesion (cm)			8.59 ± 3.47	12.71 ± 5.56		< 0.001
O-RADS (v2022) categories and specific lexicon descriptors						< 0.001
Categories	Specific lexicon descriptors					
2			100 (100)	0 (0)	100	
	Simple cyst (< 10 cm)		0	0	0	
	Unilocular, smooth, non-simple cyst/bilocular, smooth cyst (< 10 cm)		32 (100)	0 (0)	32	
	Typical benign ovarian lesion (< 10 cm)		67 (100)	0 (0)	67	
	Typical benign extraovarian lesion (any size)		1 (100)	0 (0)	1	
3			43 (100)	0 (0)	43	
	Typical benign ovarian lesion (≥ 10 cm)		5 (100)	0 (0)	5	
	Uni- or bilocular, smooth (≥ 10 cm)		9 (100)	0 (0)	9	
	Unilocular, irregular (any size)		3 (100)	0 (0)	3	
	Multilocular, smooth, < 10 cm, CS < 4		18 (100)	0 (0)	18	
	Solid lesion, ± shadowing, Smooth, any size, CS = 1		2 (100)	0 (0)	2	
	Solid lesion, shadowing, Smooth, any size, CS = 2–3		6 (100)	0 (0)	6	
4			21 (53.8)	18 (46.2)	39	
	Bilocular cyst without solid component, irregular, any size, any CS		0	0	0	
	Multilocular cyst without solid component	a. Smooth, ≥ 10 cm, CS < 4	9 (81.8)	2 (18.2)	11	
		b. Smooth, any size, CS 4	0	0	0	
		c. Irregular, any size, any CS	0 (0)	2 (100)	2	
	Unilocular cyst with solid component, < 4 pps or solid component not considered a pp; any size		4 (36.4)	7 (63.6)	11	
	Bi- or multilocular cyst with solid component, any size, CS 1–2		6 (54.5)	5 (45.5)	11	
	Solid lesion, non-shadowing, Smooth, any size, CS = 2–3		2 (50)	2 (50)	4	
5			2 (5.6)	34 (94.4)	36	
	Unilocular cyst ≥ 4 pps, any size, any CS		0 (0)	4 (100)	4	
	Bi- or multilocular cyst with solid component, any size, CS 3–4		0 (0)	6 (100)	6	
	Solid lesion, ± shadowing, Smooth, any size, CS 4		0 (0)	4 (100)	4	
	Solid lesion, irregular, any size, any CS		2 (9.5)	19 (90.5)	21	
	Ascites and/or peritoneal nodules		0 (0)	1 (100)	1	
Color score						< 0.001
1			89 (100)	0 (0)	89	
2			68 (77.3)	20 (22.7)	88	
3			8 (28.6)	20 (71.4)	28	
4			1 (7.7)	12 (92.3)	13	

O-RADS, Ovarian-Adnexal Reporting and Data Systems;* v,* version; B, Benign; M, Malignant; CS, Color Score; pps, papillary projections.

**Table 2:** Pathological types of the 218 lesions.

Pathological findings		Number
Benign		166
Endometrioma		52
Mature teratoma		39
Serous cystadenoma		29
Mucinous cystadenoma		14
Fibroma and fibrothecoma		5
Brenner tumor		1
Fallopian tube diseases		10
Other		16
Malignant		52
Serous cystadenocarcinoma		21
Mucinous cystadenocarcinoma		6
Clear cell tumor		7
Endometrioid carcinoma		3
Immature teratoma		2
Krukenberg tumor		4
Borderline tumor	Serous borderline ovarian tumor	4
	Mucinous borderline ovarian tumor	2
Other		3

### The results of these classification systems

The results of the subjective assessment and O-RADS (v1) assessment of 218 lesions were shown in Table 3, and the results of the O-RADS (v2022) assessment were shown in Table 1. The malignancy rates for the benign and malignant groups for the subjective assessment method were 4.35% and 90% respectively, and the malignancy rates for O-RADS 2, O-RADS 3, O-RADS 4 and O-RADS 5 were 0%, 0%, 36% and 94.4% for O-RADS (v1) and 0%, 0%, 46.2% and 94.4% for O-RADS (v2022) respectively.

**Table 3:** Subjective assessment of 218 lesions with O-RADS (v1) assessment results.

Group	Benign	Malignant	Malignant rate (%)	Total
Subjective assessment				
Benign group	161	7	4.4	168
Malignant group	5	45	90.0	50
O-RADS (v1)				
O-RADS 2	92	0	0.0	92
O-RADS 3	40	0	0.0	40
O-RADS 4	32	18	36.0	50
SRR < 10%	18	0		18
SRR ≥ 10%	14	18		32
O-RADS 5	2	34	94.4	36

O-RADS, Ovarian-Adnexal Reporting and Data Systems; v, version; SRR, Simple Rules Risk assessment model.

### Comparison of diagnostic efficacy between subjective assessment and O-RADS

**Figure 2:**
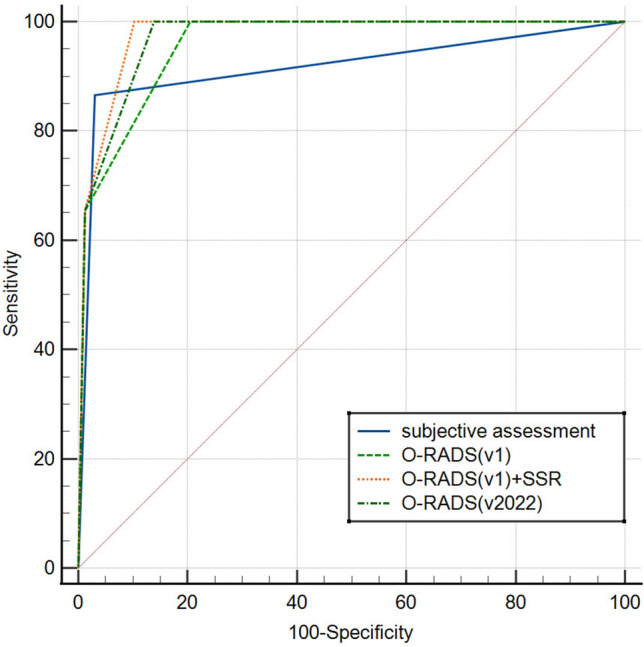
ROC curve for subjective assessment, O-RADS (v1), O-RADS (v1) combined with SRR and O-RADS (v2022).

The ROC curves showed that for O-RADS (v1), O-RADS (v2022) and O-RADS (v1) combined with SRR assessment, the best cut-off value for predicting malignancy was O-RADS 3 (Fig. 2). Of these, the AUC of O-RADS (v2022) was 0.970 (95% CI 0.938–0.988), which was not statistically significantly different from the O-RADS (v1) combined SRR assessment model with the largest AUC of 0.976 (95% CI 0.946–0.992) (*p* = 0.1534), and was significantly higher than the O-RADS (v1) (*p* = 0.0133) and subjective assessment (*p* = 0.0255). The AUC was 0.959 (95% CI 0.923–0.981) for the O-RADS (v1) and 0.918 (95% CI 0.873–0.950) for the subjective assessment, with no statistically significant difference between the two (*p* = 0.0782).

**Figure 3:**
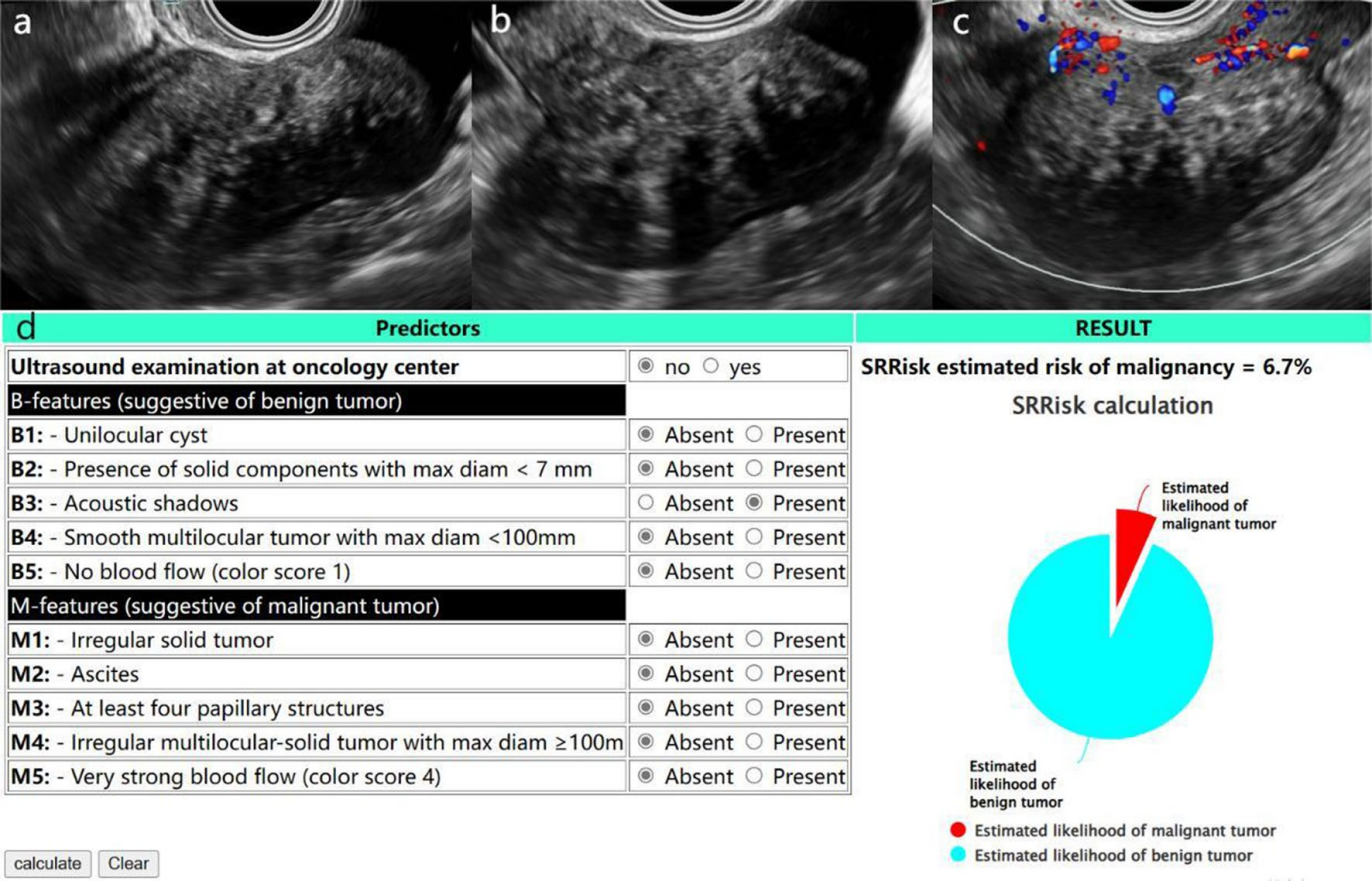
A 65-year-old woman with a fibroma of the ovary in the left adnexal region. (**a**) and (**b**) Longitudinal and transverse section of the lesion, B-mode US showed a smooth solid mass with acoustic shadowing. (**c**) Small amount of blood flow signal within the lesion (Color Score = 2). (**d**) Results of SRR assessment of the lesion. Lesions was classified as O-RADS (v1) category 4, O-RADS (v1) combined with SRR assessment and O-RADS (v2022) category 3.

**Figure 4:**
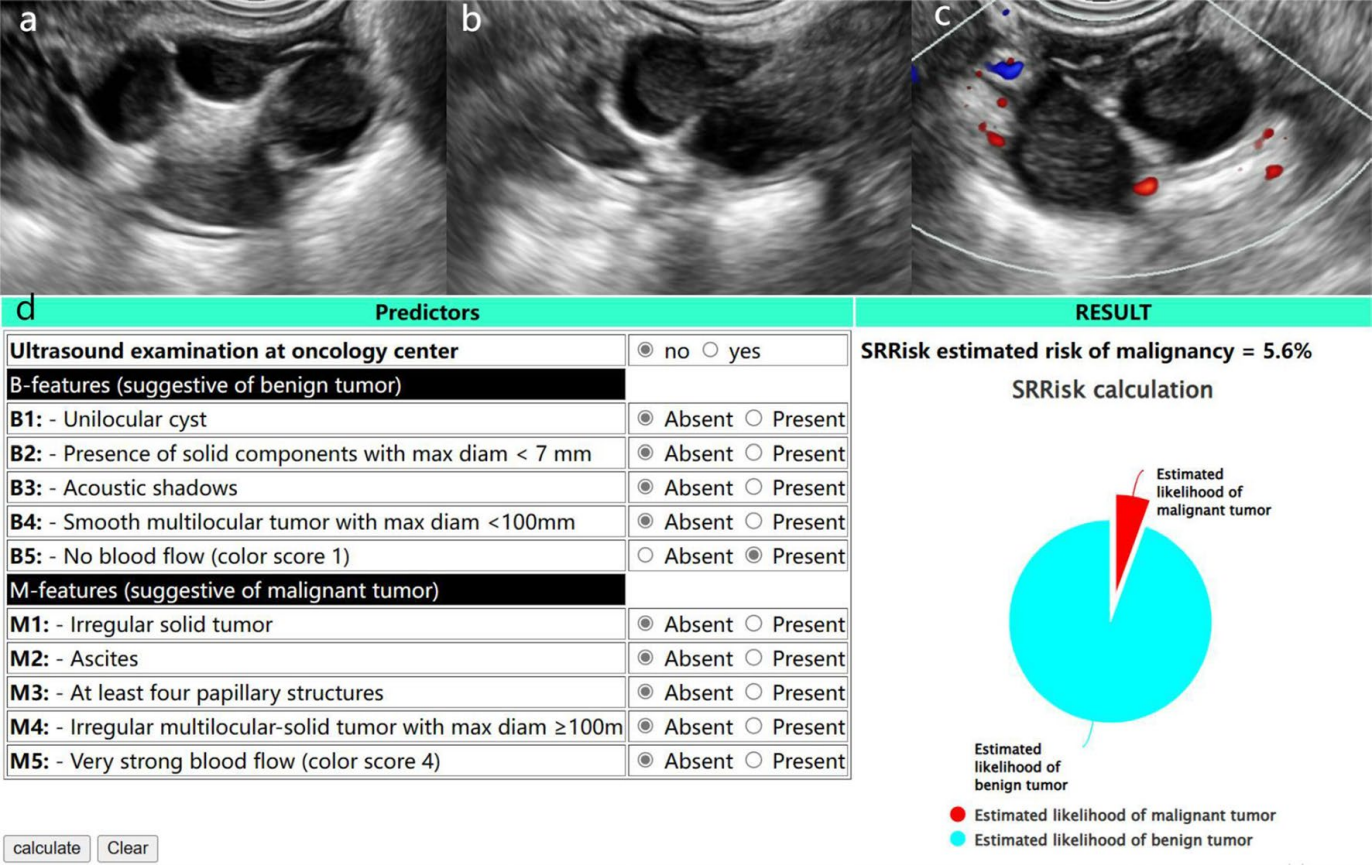
A 38-year-old woman with a Mature teratoma in the right adnexal region. (**a**) and (**b**) Longitudinal and transverse section of the lesion, B-mode US showed a multilocular cyst with a solid component (maximal diameter 4.4 cm). (**c**) No clear blood flow signal was seen within the lesion (Color Score = 1). (**d**) Results of SRR assessment of the lesion. Both O-RADS (v1) and O-RADS (v2022) of the lesion were category 4, and O-RADS (v1) combined with SRR assessment was category 3.

Of these methods, the subjective assessment had the highest diagnostic accuracy and specificity (94.5% and 97.0%, respectively), but was less sensitive than the O-RADS correlation model (86.5% vs. 100%) (Table 4). Of the lesions classified as malignant by O-RADS (v1), 18 benign lesions were successfully downgraded when assessed by combined SRR, and 11 lesions were classified as benign when assessed using O-RADS (v2022) (Figs. 3 and 4).

**Table 4:** Diagnostic efficacy of subjective assessment, O-RADS (v1), O-RADS (v1) combined with SRR and O-RADS (v2022).

Assessment method	Sensitivity (%)	Specificity (%)	Accuracy (%)	PPV (%)	AUC	P
Subjective assessment	86.5	97.0	94.5	95.8	0.918	0.0255^a^
O-RADS (v1)	100	79.5	84.4	60.5	0.959	0.0133^b^
O-RADS (v1) and SRR	100	90.4	92.7	76.5	0.976	0.0009^c^
O-RADS (v2022)	100	86.1	89.4	70.3	0.970	0.1534^d^

O-RADS, Ovarian-Adnexal Reporting and Data Systems; v, version; SRR, Simple Rules Risk assessment; PPV, Positive Predictive Value; P^a^, subjective assessment compared with O-RADS (v2022); P^b^, O-RADS (v1) compared with O-RADS (v2022); P^c^, O-RADS (v1) compared with O-RADS (v1) combined SRR; P^d^, O-RADS (v2022) compared with O-RADS (v1) combined SRR.

## Discussion

As a common gynecological tumor, it is extremely important that ovarian cancer is accurately assessed preoperatively^14^. As a newly proposed US classification system, most studies are still in the process of validating the diagnostic efficacy of O-RADS and its observer agreement^15–21^. Currently, subjective assessment by senior sonologists is considered the most accurate method for diagnosing AMs^22^. In this study, we evaluated the performance of the O-RADS series of models in the preoperative identification of benign and malignant AMs and compared them separately with other commonly used clinical assessment methods. The overall results indicated that the O-RADS, particularly the O-RADS (v2022), has a high diagnostic efficacy and can assist the sonologists in the accurate preoperative assessment of benign and malignant AMs.

As in previous studies, there were statistically significant differences between benign and malignant AMs in this study in terms of patient age, lesion size, lesion type, and lesion blood flow score (all *P* < 0.001)^17,21^. Meanwhile, Di Legge et al.^23^ and Bruno et al.^24^ mentioned that even for lesions of small dimension, some ultrasound features such as irregular contour, absence of acoustic shadowing, vascularized solid areas, ≥ 1 papillae, vascularised septum and moderate-severe ascites, also play a role in the differentiation of benign and malignant lesions. The results of this study showed that the malignancy rates for O-RADS 2, O-RADS 3, O-RADS 4 and O-RADS 5 were 0%, 0%, 36% and 94.4% for O-RADS (v1) and 0%, 0%, 46.2% and 94.4% for O-RADS (v2022) respectively, with the malignancy rate of O-RADS 3 being less than the 1–10% provided in the guidelines^7^. Analysis of the reasons for this may be related to the small sample size included in this study and the small number of pathological types involved.

The low specificity of O-RADS (v1) has received a lot of attention^10,19,21^. Lan Cao et al.^10^ suggested that the diagnostic accuracy and specificity of O-RADS (v1) could be effectively improved if multilocular cysts and smooth solid masses in the 4 categories of O-RADS (v1) were classified as benign. Based on existing studies, O-RADS (v2022) provides a more specific classification of multilocular cysts and smooth solid masses in the O-RADS category 4^10,19,21^. It also downgrades smooth bilocular cyst, which is ≥ 10 cm, and smooth solid lesion with acoustic shadow and color score (CS) of 2–3 to category 3. During the study, 11 benign lesions were successfully downgraded when classified using O-RADS (v2022), with significant improvements in diagnostic accuracy (84.4–89.4%) and specificity (79.5–86.1%) without altering sensitivity. These 11 lesions included three ovarian fibromas and one Brenner tumor (with acoustic shadow, CS = 2). Ovarian fibromas are the most common type of sex cord-stromal tumors and the lesions tend to present as smooth solid masses with acoustic shadowing and a small or moderate amount of blood flow (CS = 2–3)^25^. According to the O-RADS (v1) classification criteria, the lesions are mostly classified as O-RADS 4^7^. When > O-RADS 3 is used as a predictor of malignancy, the lesions are often classified in the malignant category. The O-RADS (v2022) classification system classifies smooth solid lesions with acoustic shadowing and a 2–3 color score as category 3. When using this classification method, some ovarian fibromas and fibrothecomas with typical US features can be correctly classified as benign, effectively avoiding unnecessary surgery in some patients.

Lan Cao et al.^10^ proposed that the O-RADS (v1) category 4 of lesions are similar to the uncertain category in the IOTA SRs. To calculate the specific malignancy risk of lesions in the SRs model, the IOTA group developed the SRR assessment model in 2016^12^. Numerous studies have confirmed that IOTA SRR assessment, ADNEX model and ORADS can help in the differentiation of benign and malignant masses^15–21,26^. In this study, the category 4 of O-RADS (v1) lesions were assessed for malignancy risk using SRR assessment and downgraded using 10% as the cutoff value, resulting in a combined assessment model with the largest AUC (0.976, 95% CI 0.946–0.992). However, the AUC of the combined model was not statistically significantly different from the AUC of O-RADS (v2022) (*p* = 0.1534). Thanks to its higher sensitivity, O-RADS (v1) is able to detect malignancies sensitively, minimising the occurrence of missed diagnoses, but its lower specificity may allow patients with AMs to be over-treated in the clinic^15,19,21^. Similar to the O-RADS (v2022) classification system, when assessed in combination with the SRR assessment, the specificity of the O-RADS (v1) was significantly improved (79.5–90.4, *p* = 0.006) without reducing diagnostic sensitivity. However, this is a single-centre study and much research is needed to determine the diagnostic efficacy of O-RADS (v1) combined with SRR assessment and how to further improve the specificity of O-RADS (v1) diagnosis.

A study by Moro et al*.*^27^ mentioned that serous borderline ovarian tumor showed an overlaping ultrasound appearance with non-invasive low-grade serous ovarian carcinoma, both presenting as cysts with papillary projections. However, unlike ovarian cancer, the prognosis for borderline tumors is relatively good, and women of fertile age can be treated with fertility-sparing surgery^28^. Therefore, it is extremely important and necessary to accurately distinguish borderline tumors from ovarian cancer before surgery. A total of 6 borderline tumors (4 Serous and 2 mucinous ovarian borderline tumors) were enrolled in the present study, and considering that the patients were in Stage I, and all were women of fertile age (range, 22–34 years), the surgical approach used for this group of patients was fertility-sparing surgery. Moro et al*.*^27^ proposed that the serous borderline ovarian tumor were described as unilocular-solid or as multilocular-solid with solid papillary projection. Meanwhile, another study by Moro *et al*^29^ suggested that a multilocular cyst with 2–10 locules is representative of a benign cystadenoma, whereas a multilocular cyst with > 10 locules is indicative of a gastrointestinal (GI)-type borderline tumor. The borderline tumors included in this study exhibited multilocular cyst (two cases, maximum diameter > 10 cm and > 10 locules) or multilocular cyst with solid component on ultrasound, and such lesions were classified as O-RADS categories 4 and 5 for both O-RADS (v1) and O-RADS (v2022) assessments, and lesions classified as category 4 failed to be downgraded for combined SRR assessment. Ludovisi et al*.*^30^ described the serous surface papillary borderline ovarian tumors (SSPBOTs) a rare morphologic variant of serous ovarian tumors that are typically confined to the ovarian surface, as irregular solid lesions surrounding normal ovarian parenchima. There were no SSPBOTs in the cases included in this study, but according to the O-RADS classification guidelines, such lesions met the classification criteria of O-RADS 5 in both O-RADS (v1) and O-RADS (v2022). Considering that the biological behavior of borderline tumors is intermediate between benign and malignant^31^, giving them a higher assessment of malignant risk can draw the attention of clinicians to avoid delaying patient treatment. However, the ability of the O-RADS classification system to identify borderline tumors is indeed limited, and which of the lesions assessed to be at moderate or high risk of malignancy are borderline tumors will have to be subjectively evaluated by experienced sonologists, which is a limitation of the O-RADS classification system that should be improved in subsequent studies.

The main strength of this study is that the results of subjective assessment and O-RADS (v1) assessment were collected prospectively and pathological results were available for all lesions. However, the O-RADS (v2022) classification results in this study were obtained from retrospective analysis of lesions, and the small sample size and single-centre nature of this study may lead to limitations in the wider application of the findings. In addition, all patients included in this study were those with obtainable pathology after surgery for AMs. Patients in both O-RADS 0 and O-RADS 1 categories were not included, which may result in selection bias and overestimation of PPV.

In summary, the O-RADS series models have good diagnostic performance for AMs. Among them, O-RADS (v2022) has higher diagnostic efficacy and diagnostic specificity than O-RADS (v1). However, when O-RADS (v1) is combined with SRR assessment, its diagnostic accuracy and specificity can be further improved.

## Data Availability

The data that support the findings of this study are available from the corresponding author upon reasonable request.
